# Heterogeneous Pattern of Retinal Nerve Fiber Layer in Multiple Sclerosis. High Resolution Optical Coherence Tomography: Potential and Limitations

**DOI:** 10.1371/journal.pone.0013877

**Published:** 2010-11-08

**Authors:** Nermin Serbecic, Fahmy Aboul-Enein, Sven C. Beutelspacher, Martin Graf, Karl Kircher, Wolfgang Geitzenauer, Werner Brannath, Priska Lang, Wolfgang Kristoferitsch, Hans Lassmann, Andreas Reitner, Ursula Schmidt-Erfurth

**Affiliations:** 1 Department of Ophthalmology, Medical University of Vienna, Vienna, Austria; 2 Department of Neurology, SMZ-Ost Donauspital, Vienna, Austria; 3 Department of Ophthalmology, Faculty of Medicine Mannheim, University of Heidelberg, Mannheim, Germany; 4 Department of Biostatistics, Medical University of Vienna, Vienna, Austria; 5 Brain Research Centre, Medical University of Vienna, Vienna, Austria; Julius-Maximilians-Universität Würzburg, Germany

## Abstract

**Background:**

Recently the reduction of the retinal nerve fibre layer (RNFL) was suggested to be associated with diffuse axonal damage in the whole CNS of multiple sclerosis (MS) patients. However, several points are still under discussion. (1) Is high resolution optical coherence tomography (OCT) required to detect the partly very subtle RNFL changes seen in MS patients? (2) Can a reduction of RNFL be detected in all MS patients, even in early disease courses and in all MS subtypes? (3) Does an optic neuritis (ON) or focal lesions along the visual pathways, which are both very common in MS, limit the predication of diffuse axonal degeneration in the whole CNS? The purpose of our study was to determine the baseline characteristics of clinical definite relapsing-remitting (RRMS) and secondary progressive (SPMS) MS patients with high resolution OCT technique.

**Methodology:**

Forty-two RRMS and 17 SPMS patients with and without history of uni- or bilateral ON, and 59 age- and sex-matched healthy controls were analysed prospectively with the high resolution spectral-domain OCT device (SD-OCT) using the Spectralis 3.5mm circle scan protocol with locked reference images and eye tracking mode. Furthermore we performed tests for visual and contrast acuity and sensitivity (ETDRS, Sloan and Pelli-Robson-charts), for color vision (Lanthony D-15), the Humphrey visual field and visual evoked potential testing (VEP).

**Principal Findings:**

All 4 groups (RRMS and SPMS with or without ON) showed significantly reduced RNFL globally, or at least in one of the peripapillary sectors compared to age-/sex-matched healthy controls. In patients with previous ON additional RNFL reduction was found. However, in many RRMS patients the RNFL was found within normal range. We found no correlation between RNFL reduction and disease duration (range 9–540 months).

**Conclusions:**

RNFL baseline characteristics of RRMS and SPMS are heterogeneous (range from normal to markedly reduced levels).

## Introduction

A recently broadly discussed hypothesis is that analysis of the retina nerve fibre layer (RNFL) may be useful to track degenerative processes in the central nervous system (CNS) [1]–[2]. In previous studies on patients with multiple sclerosis (MS) axonal loss in the RNFL was found to be correlated to visual impairment, and interestingly to disability scores composed of other than visual CNS dysfunctions (e.g. motor system) [3]. These findings suggested that in MS the damage affecting the entire CNS may be visualized by scanning the damage in the RNFL and in the optic discs. However, since standard time-domain optical coherence tomography (TD-OCT) was developed to detect or monitor rather gross pathologies such as glaucoma, retinal tumours, macula oedema, it is currently unclear if RNFL changes of some micrometers are even detectable, and if so reliably measurable with TD-OCT [4], [5]. Apart from that, several other issues must be clarified. First, it is conceivable that prior clinically silent or apparent episodes of optic neuritis or focal lesions along the visual pathway, which are both very common in MS, may also lead to RNFL reduction. If so, a predication of global and more subtle progressive axonal degeneration would be very difficult. Secondly, the true extent of focal MS lesions can hardly be specified, when they were clinically silent, ignored or at least not remembered by the patients, or were undetectable by magnetic resonance imaging (MRI) or spectroscopy [6]–[8]. Thirdly, technical limitations must be kept in mind. As prolonged examination time and poor fixation result in motion artefacts and decentred image scans, the use of TD-OCT seems restricted for follow-up examinations [9]–[11]. In contrast the newly developed high-resolution spectral-domain OCT (SD-OCT) imaging technology overcomes these technical limitations of TD-OCT markedly. With SD-OCT ultra-high speed retina scanning image artefacts due to dispositioning of the scan detector, eye movements or poor fixation can be avoided [11]. Most importantly, identical scanning locations can be adjusted over time, and thus, follow-up examinations easily performed [10].

In our study we used a new high-resolution SD-OCT to analyse the RNFL in a large cohort of well classified patients with clinically definite relapsing-remitting (RRMS) or secondary progressive MS (SPMS) and healthy age- and sex matched controls to RNFL characteristics at baseline [12]. Additionally, visual and contrast sensitivity scores, color vision, visual field testing, and visual evoked potentials were performed. Most importantly, in our study results of MS eyes with and without history of ON were distinguished, and separately analysed.

## Methods

### Participants

The study was approved by the local Ethics Committee (Commission of Medical Ethics of Vienna; Ethic Approval/Registration Number: EK-08-028-0308 and Ethical commission of the Medical University of Vienna; Ethic Approval/Registration Number: 414/2008). Informed written consent was obtained from all patients and volunteers before study entry.

We studied 59 patients with clinically definite MS, and 59 sex- and age matched healthy controls [12]. MS diagnosis was based on clinical course, MRI and on cerebrospinal fluid (CSF) analysis. Oligoclonal bands were found in all MS CSF samples. Forty-two MS patients followed a relapsing-remitting course (RRMS) and 17 MS patients a secondary progressive course (SPMS). In 5 cases with recurrent optic neuritis and 3 with myelitis the analysed sera were negative for aquaporin-4 autoantibodies [13]. Thirteen RRMS and 11 SPMS had a history of prior ON. A summary of all MS patients and controls is given in table 1.

**Table 1:** 

		MS cases with and without Optic Neuritis (ON)				MS cases with Optic Neuritis (ON)			
	No	age (years) [range]	disease duration (months) [range]	all Relapses	No	age (years) [range]	disease duration (months) [range]	all Relapses	all ON
**Controls**	59	38.9±1.35[21 to 67.0]	-	-	-	-	-	-	-
**RRMS & SPMS,** **total**	59	39±1.4[19.8 to 68.0]	121.1±14.2	4.5±0.4[1 to 19]	24	40.7±2.4[19.8 to 68.0]	161.4±21.1[25.0 to 350.0]	4.5±0.4[1 to 19]	1.8±0.2[1 to 4]
**RRMS, total**	42	37.6±1.7[19.8 to 68.0]	90.3±15.2[9.0 to 540.0]	3.8±0.3[1 to 10]	13	38.8±3.8[19.8 to 68.0]	117.3±21.9[25.0 to 324.0]	4.6±0.6[2 to 9]	2.0±0.3[1 to 4]
**RRMS, female**	31	37.1±2.2[19.8 to 68.0]	101.2±18.8[14.0 to 540.0]	4.1±0.4[2 to 9]	12	38.9±3.8[19.8 to 68.0]	117.3±21.9[25.0 to 324.0]	4.6±0.6[2 to 9]	2.1±0.3[1 to 4]
**RRMS, male**	11	38.3±2.3[25.0 to 52.0]	56.6±22.4[9.0 to 240.0]	3.0±0.7[1 to 10]	1	33	25	2	1
**SPMS, total**	17	43.0±2.2[26.0 to 56.0]	197.2±23.8[40.0 to 350.0],	3.3±1.0[1 to 19]	11	42.9±2.8[26.0 to 53.5]	213.5±32.3[40.0 to 350.0]	5.8±0.8[3 to 12]	1.5±0.2[1 to 2]
**SPMS, female**	10	45.0±2.8[27.0 to 56.0]	210.4±34.2[40.0 to 350.0],	3.0±0.7[1 to 10]	6	46.6±2.7[35.8 to 53.5]	236.8±50.8[40.0 to 350.0]	5.8±1.3[3 to 12]	1.7±0.2[1 to 2]
**SPMS, male**	7	40.9±3.7[26.0 to 52.0]	148.4±32.7[78.0 to 282.0]	6.5±1.6[3 to 19]	5	38.5±4.9[26.0 to 52.0]	102.±38.9[102.0 to 282.0]	5.8±1.2[3 to 10]	1.4±0.2[1 to 2]

### Exclusion criteria

Patients with other diseases that reduce RNFL thickness such as glaucoma, anterior ischemic optic neuropathy, high myopia, and congenital abnormalities of the optic nerves were excluded.

Baseline clinical neurological examinations, visual evoked potentials (VEP), and ophthalmologic examinations were performed within 7 days. The patients' history and available medical records were carefully reviewed for previous ON. The diagnosis of ON was based on clinical findings, which included the presence of decreased visual acuity, a visual field defect, colour vision loss, relative afferent pupil defect, and a compatible fundus examination. No included patient had an ON within 12 months prior to the beginning of the study.

### Visual function testing

Visual function testing (best corrected visual acuity and sensitivity) was performed in MS patients and controls using low-contrast Sloan letter charts (retro-illuminated version, Precision Vision, IL) at 10% and at 5% low-contrast levels and Pelli-Robson contrast sensitivity chart and at full contrast (100% ETDRS 2000 chart, testing high-contrast visual acuity). All testing was performed monocularly and with both eyes together. If one eye was visually worse, this eye was tested first followed by the better eye; binocular testing was performed last. The results were recorded as the number of letters correctly identified (with a maximum of 60 letters per chart, each line  = 5 letters for the ETDRS and Sloan chart and a maximum of 48 letters with 6 letters each line for the Pelli-Robson chart). Both patients and controls were asked to use their glasses/contact lenses to correct for the testing.

### Visual Field Analysis

Visual field analysis was obtained with Humphrey perimetry using the SITA algorithm (Zeiss Meditech, Dublin, CA). For the purposes of comparing visual field function (measured in decibels to the mean RNFL thickness (measured in micrometers), we used central 30-2, full-threshold strategy testing [14].

### RNFL Measurement

The high resolution SD-OCT uses a scanning superluminescence diode to emit a scan beam with a wavelength of 870 nm to provide up to 40.000 A scans/sec with a depth resolution of 7 µm in tissue and a transversal resolution of 14 µm of images of ocular microstructures (Heidelberg Engineering, Heidelberg, Germany, Spectralis software version 4.0.3.0, Eye Explorer Software 1.6.1.0). The instrument combines OCT technology with a confocal Scanning Laser Ophthalmoscope (Heidelberg Engineering, Heidelberg, Germany), which provides a reference fundus image. By means of eye tracking (TrueTrac™) each peripapillary OCT scan will be registered and locked to a reference image. OCT software can identify previous scan locations and “guide” the OCT laser beam to scan the same location again. For this purpose, eye tracking and the high scanning speed is supposed to reduce artefacts resulting from eye movement. For OCT scanning, the high-resolution SD-OCT provides an Automatic Real-Time averaging mode (ART mode) which when activated enables the operator to adjust the number of obtained frames (B scans) to average images for increased image quality. In this study, this was achieved by a total of 16 frames (maximum available number of image frames) of the same scanning location during the peripapillary scanning process thereby optimizing the signal to noise ratio and image quality significantly. Examination time is not the same as scanning speed. Although scanning speed of the high-resolution SD-OCT for a B-scan image is extremely fast, performing up to 40.000A scans/sec, B scans, however, will only be performed if the eye tracking software recognizes the exact scanning position in the fundus image. Scans were acquired in the high-resolution acquisition mode allowing a more detailed differentiation of retinal layers, with pupil dilation.

An internal fixation light was used to centre the scanning area on the disc. To obtain excellent centring, the incorporated circle-shaped target area was placed on the optic disc and performed scans were obtained within a single session on all eyes. Scans were performed with activated ART mode during measurements with locked target area over the fixed optic disc reference image for several scans. RNFL scans were performed several times by one operator (N.S.) within one session until at least 3 high-quality scans were achieved and used for further analysis.

**Figure 1:**
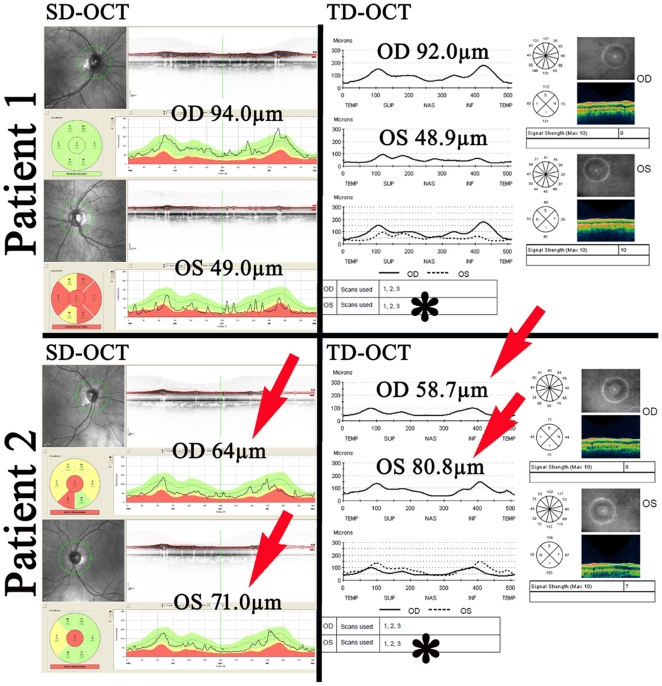
Comparison of the high resolution SD-OCT and standard TD-OCT. Figure 1 shows high resolution SD-OCT scans (left column) and standard TD-OCT scans (right column) of 2 MS patients. Patient 1, 34 year old female, RRMS, disease duration 15years, prior ON (left eye, 2 years after disease onset). Patient 2, SPMS, 47 year old male, SPMS, disease duration 20 years, no prior ON. Measurements of RNFL were comparable in patient 1 but differed about 5.3 µm (OD, right eye) and 9.8 µm (OS, left eye) in patient 2 (red arrows, lower row). Baseline/reference scans of standard TD-OCT are averaged out of 3 sequential scans (asterisks) with partly substantial differences of each single scan. Low scanning speed and prolonged examination time of standard TD-OCT can often not compensate low image resolution despite good centering around the optic nerve as a result of poor fixation and motion artefacts. In contrast, with high resolution SD-OCT reliable and properly centered baseline and follow up scans can be achieved with ultrahigh speed scanning and an eye tracking mode. Moreover, with high resolution SD-OCT previous scan locations can be identified and guide the laser beam to identical scan positions repeatedly.

Furthermore, signal strength has been shown to affect RNFL thickness measurements using conventional Stratus OCT [15]–[16] (Figure 1, manuscript in preparation). Therefore, scans with low quality and failing RNFL segmentation were excluded. Measurements were repeated until excellent quality was achieved. Criteria for determining scan quality include (1) a clear fundus image before and during image acquisition, (2) absence of scan or algorithm failures, (3) even and dense grey scale saturation throughout all retinal layers with dense grey visible in the RPE, and (4) the RNFL visible without missing or blank areas and a continuous scan pattern.

### Visual evoked potentials (VEP)

VEP were performed with essentially the same protocol as described in very detail previously [17].

### Statistics

**Figure 2:**
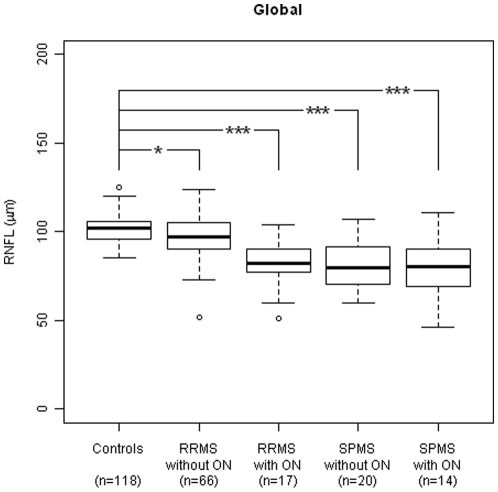
Global reduction of RNFL. *p<0.05; **p<0.005; ***p<0.0001.

**Figure 3:**
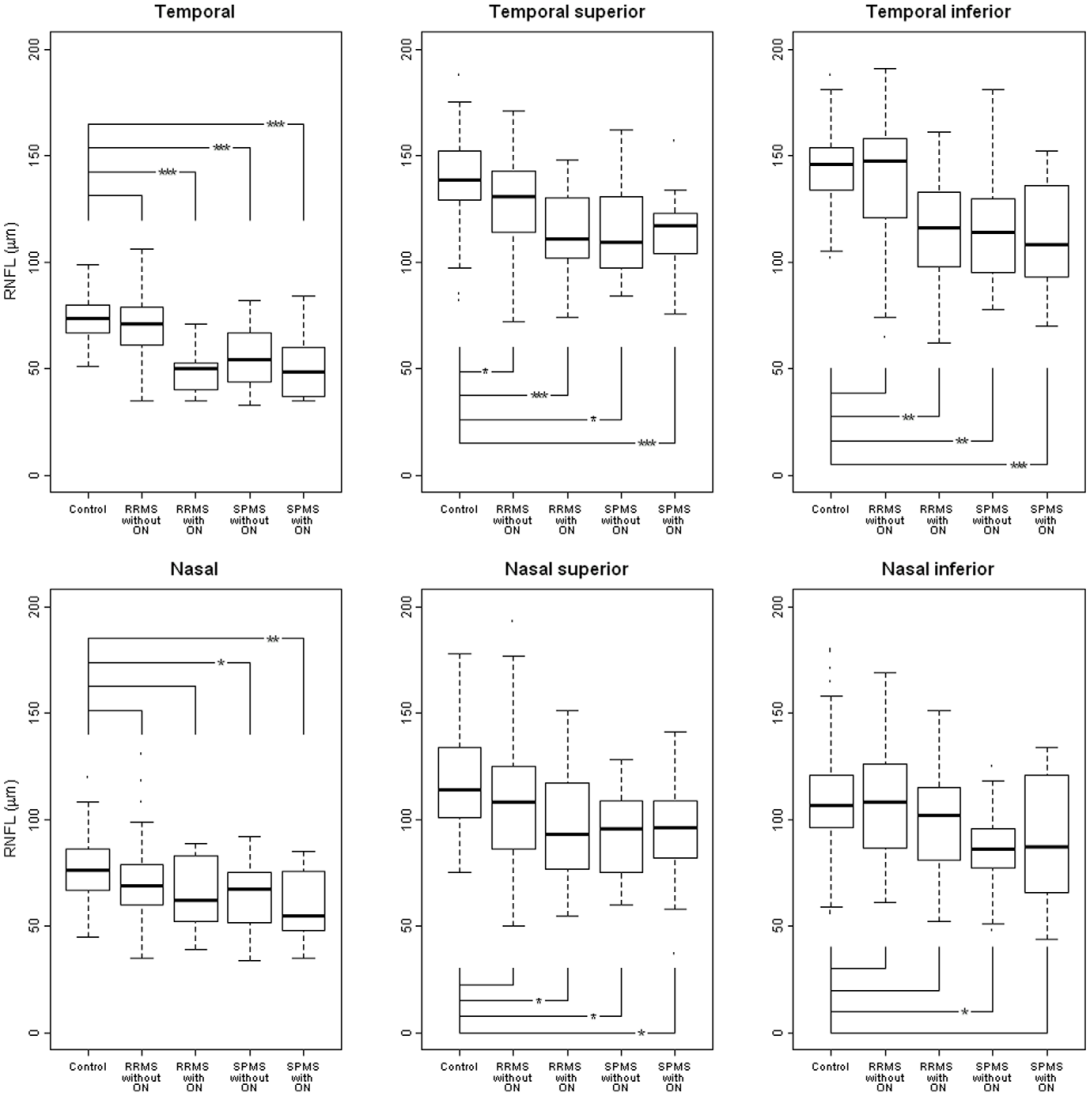
Segmental reduction of RNFL. *p<0.05; **p<0.005; ***p<0.0001.

Qualitative variables were summarized by minimum, maximum, mean and standard deviation, and by frequency tables. P-values below 0.05 were considered statistically significant. We performed the Bonferroni-Holm procedure for the comparisons in Figure 2 and Figure 3 (separately for each figure) and present the corresponding multiplicity adjusted p-values. Multiplicity adjusted p-values are larger than the raw p-values and can directly be compared to the 5% significance level. The model assumptions for the random effect models are investigated by residual plots and q-q-plots. All analyses were performed with the statistical software package R version 2.9.0, in particular, the R-package nlme.

We compared the influence of disease status (RRMS versus SPMS) and prior optic neuritis (ON) on RNFL by forming four different MS-groups, namely, RRMS eyes with and without ON, SPMS eyes with and without ON (groups 2 and 4, respectively) and age/sex-matched healthy controls. We analyzed the overall mean (global) and sector wise RNFL values by a random effect model with two nested random factors accounting for a correlation of measurements within matched pairs and patients. Similar analyses of global measurements were done within the group of patients with one ON-free and one ON fellow eye.

We furthermore investigated the influence of age at the time of OCT measurement, MS group (RRMS and SPMS with/without ON), and disease duration (change of RNFL per decade) in a mixed model for patient eyes including the three fold interaction term for sector, disease group and disease duration. The main intention of this analysis was to investigate whether disease duration influences the RNFL in MS patients with ‘ON free eyes’ or not. The overall effect of a prior ON episode on the mean RNFL is estimated by a random effect models with ON (yes/no) as single fixed factor and the patient as random factor.

The correlation of full-contrast visual acuity (EDTRS, Pelli-Robson and Sloan charts), Lanthony desaturated colour vision test, and the “Mean Deviation” of the Humphrey visual field analysis with RNFL was investigated by mixed models where each of the tests was taken as a single fixed effect and age at OCT as additional random factor. The mean RNFL loss associated with a one line decrease in EDTRS, SLOAN 5% and 10% (loss of 5 letters) and in the PR scale (loss of 6 letters) is estimated by the mixed model of the sector-wise data when there is a no significant interaction between sector and visual test and otherwise from a mixed-effect model for the mean ( = global, figure 3) RNFL. The correlation between RNFL and “Mean deviation” (MD) in visual field analysis or P-100 latencies was investigated by similar mixed models. We furthermore investigated by a logistic regression analysis with a robust variance estimate (generalized estimation equation approach) the influence of the RNFL on the likelihood for a P100 below 118 ms and for a MD variation ≥ ±2.5 decibel.

To verify the company standard values, estimates and 95% confidence intervals for the mean RNFL were determined from the control group with variance component models for each sector. The variance component models were performed to account for the correlation between the two eyes of a control person. Company standard values are compared to the 95% intervals.

## Results

### Company standard values

The 95% confidence intervals of RNFL measurements for controls and different disease groups and the standard values of the OCT company are given in Table 2.

**Table 2:** 95% confidence intervals (upper and lower values) and estimates (middle value) for RNFL in the different sectors for different disease and control groups.

Sector	RRMS withoutON	RRMS withON	SPMS without ON	SPMS withON	Control eyes	Heidelberg Spectralisvalues
Meanof all sectors ± SD	91.2995.6299.95	71.6880.4189.14	74.3483.2092.05	68.7379.1089.46	99.42101.51103.60	97
T	65.7070.4775.23	42.8250.3057.77	48.6157.1065.58	40.9450.0759.19	71.1073.4375.76	74
TI	132.64139.94 147.24	100.15114.28128.41	103.81 119.64 135.47	97.33111.90 126.46	140.13144.09148.07	**141**
NI	100.22 108.75 117.29	80.9496.38111.82	73.7285.4597.17	65.6087.80110.00	103.10108.70114.29	105
N	64.2269.4974.77	55.9165.0774.23	55.7764.7573.73	48.9358.5268.12	74.2477.4580.66	**72**
NS	98.038 108.23 118.42	77.1894.18111.19	84.6494.52104.39	79.8497.36114.89	113.21118.50123.79	**105**
TS	121.16 128.02 134.89	98.54112.79 127.03	104.33 118.98 133.63	103.88 115.50 127.12	134.22138.43142.65	**134**

The last column gives standard values of the company. Underestimated sectors TI, N, NS, TS are shown in bold letters. SD  =  Standard deviation.

The company standard values are well covered by the 95% confidence intervals of the control group only in sectors T and NI but not in sectors TI, TS, N and NS were the company standard value is at the border (sector TI) or quite below the lower confidence limits (sectors TS, N, NS, see Table 2). Using company standards as opposed to a real matched control group may therefore result in an underestimation of RNFL alterations in MS patients. This notion is also supported by our findings, showing no significant differences between controls and RRMS patients without ON, when company standards are used.

### Differences between RRMS, SPMS and age-/sex-matched controls

Residual and q-q-plots were found to be in good agreement with the model assumptions of the random effect models. Figures 2 and 3 give boxplots and sample sizes of RNFL measurements for each of the four MS subgroups (group 1, RRMS without ON; group 2, RRMS with ON; group 3, SPMS without ON and group 4, SPMS with ON) and age and sex matched controls. The main findings may be summarized briefly as follows:

All 4 groups showed significantly reduced RNFL globally compared to controls, and in at least in one of the peripapillary sectors. In RRMS and SPMS patients without ON the mean RNFL (global) was found reduced significantly (group 1, p = 0.01; group 3, p<0.0001; figure 2). The peripapillary sectors T, TI and TS of group 2-4 (p<0.05; figure 3), and in sectors N, NI and NS of group 3-4 (p<0.05; figure 3) showed reduced RNFL compared to controls. In RRMS without ON (group 1, figure 3) the RNFL was significantly reduced in TS (p = 0.04) only. A subgroup analysis of RRMS and SPMS with one unaffected and one affected optic nerve, showed significant reduced global RNFL in both eyes (RRMS ON diseased eyes, p<0.0001; RRMS, ON ‘free’ eyes, p = 0.02; SPMS ON diseased eyes, p<0.0001; SPMS, ON ‘free’ eyes, p = 0.001).

### Influence of disease duration or subtype of MS

Neither age nor disease duration nor MS subtype showed statistical significant correlations. The mixed model for the mean nerve thickness with ON (yes/no) as single fixed factor indicates an average loss in nerve thickness associated with a prior ON episode by −11.9 µm (95% confidence interval: −16.0 to −7.7).

### Correlation of visual tests and RNFL

All visual and contrast acuity and sensitivity tests (ETDRS, Sloan and Pelli-Robson-charts), the colour vision test (Lanthony D-15) and the Humphrey visual field analysis had statistically significant correlations to the RNFL measurements (all p<0.001).

### Correlation of VEP and RNFL

Global RNFL reduction and RNFL reduction in the sectors T, TI and TS were found correlated to pathologic, increased P-100 latencies (p<0.0001). An overall association of P-100 values to RNFL was confirmed by the mixed model analysis for the average RNFL all sectors (p<0.0001; slope −0.36; 95% CI for slope −0.40 to −0.32) as well as the robust logistic regression analysis for the influence of the average RNFL on the likelihood for a P-100 latency below 118 ms (p = 0.0003; odd ratio, 0.94; 95% CI for the odd ratio, 0.91 and 0.96).

## Discussion

Purpose of our study was to define RNFL characteristics of RRMS and SPMS patients at baseline with high resolution SD-OCT technique.

Obviously, ON and technical limitations are the major factors which may influence RNFL measurements. ON is a very common, often initial symptom of MS, and leads to RNFL reduction, globally or segmentally depending on the exact location and extent of lesions within the optic nerves. Thus, most attention must be paid to prior episodes of ON, when RNFL measurements should serve for monitoring slow progressive axonal degeneration. The dilemma is that careful and detailed clinical examination and history taking, neuro-ophalmologic examination, VEP and MRI cannot completely rule out focal lesions along the optic nerves or visual pathways. After years patients are sometimes not able to remember their prior ON, even if the ON was severe but achieved full recovery. Clinical examination, VEP and MRI may be normal. In our study pathologic VEP were found pathologic in 5 out of 13 RRMS patients (8 out of 16 eyes) with prior ON only. Minimal changes within the optic nerves, such as (minimal) demyelination, remyelination and gliosis, can only be verified by biopsy, and their influence on RNFL reduction will remain unclear even with improved OCT technique. However, whether the lesion load (or very subtle MRI changes only detectable with improved MR technique e.g. [6], [7]) from the *lateral geniculate* nuclei to the visual cortex areas consistently correlates to RNFL changes (manuscript in preparation), and if so, whether these lesions may affect the optic nerves and the RNFL due to retrograde transsynaptic axonal degeneration remains under discussion (for review see [18]). To the best of our knowledge, evidence for retrograde transsynaptic axonal degeneration along the visual pathways in humans is still lacking.

In most of the MS cases, the progressive axonal changes may be elusive in short but obvious in long observations periods. To date, no reliable tool exists to monitor diffuse axonal degeneration during short disease intervals in MS patients who mostly follow a primary relapsing-remitting course with clinical stable intervals over months, years or sometimes even decades. Only rarely, the disease follows a (fast) progressive course from the beginning. Hence, diffuse axonal changes in the whole CNS and, if this hypothesis is right, RNFL reduction may be expected to be very subtle in most of the MS patients [2], [5], [18]. Moreover, reduced image quality may cause differing results on RNFL measurements within one single session as well as between follow-up visits and make standardized comparison per se impossible [11]. Decentring, poor fixation and eye movements must be avoided strictly during scan acquisition as the RNFL is not homogeneous but thicker around the optic disc [10]. The patients must be alert and able to focus a central target to avoid decentring and to keep scan acquisition time short [1], [10]–[12]. However, concentration and visual acuity are often impaired in MS patients [19]. For that reasons we and others propose minimal technical requirements to achieve high resolution baseline and reproducible follow up scans [4], [5]. To the best of our knowledge, no other study of MS patients performed with the just recently available new high-resolution SD-OCT device has been published.

In our cohorts of 42 RRMS and 17 SPMS patients a significant RNFL reduction was found in all groups, with and without prior episodes of ON.

In RRMS without ON, the RNFL reduction was observed in the sector TS (p = 0.04;) and was only evident when compared with healthy age/sex-matched controls (group 1, figure 3). The OCT device lacking a normative database, did not detect a significant reduction in RNFL in the RRMS without ON (Table 2). The inter-individual variation of RNFL is known to be high in the normal population [1], [18]. Consistent with previous studies we found a significant loss of RNFL in RRMS patients with prior uni,- or bilateral ON [20]. Axonal loss was evident in all peripapillary sectors except for the NI sector as compared to the fellow eye and disease free controls.

SPMS patients without previous ON revealed a marked RNFL reduction in both eyes. In these patients the RNFL was found more reduced than in age- and sex-matched controls. In SPMS patients without prior ON RNFL thinning might also be of functional importance since VEP were found pathologic in 83%, high-contrast visual acuity (EDTRS) in 50% and colour vision testing in 33% of 12 investigated eyes.

### Conclusions

RNFL reduction can be found in RRMS and to higher extent in SPMS patients. In addition we found marked RNFL reduction in RRMS and SPMS with ON. Underlying mechanisms, which cause the RNFL reduction apart from ON cannot be distinguished with OCT. RNFL reduction may be caused by secondary axonal degeneration due to distant focal lesions in the visual pathways or by diffuse progressive axonal degeneration due to a compartmentalized ongoing CNS inflammation. Thus it remains unclear if RNFL analysis is an appropriate method for monitoring disease progression. Prerequisite for the interpretations of OCT-investigations are standardized technical requirements [4], [5], [18].
